# Interplay between Sublethal Aminoglycosides and Quorum Sensing: Consequences on Survival in *V. cholerae*

**DOI:** 10.3390/cells10113227

**Published:** 2021-11-18

**Authors:** André Carvalho, Evelyne Krin, Chloé Korlowski, Didier Mazel, Zeynep Baharoglu

**Affiliations:** 1 Unité Plasticité du Génome Bactérien, Institut Pasteur, UMR3525 CNRS, 75015 Paris, France; andre-filipe.paulino-carvalho@pasteur.fr (A.C.); evelyne.krin@pasteur.fr (E.K.); chloe.korlowski@gmail.com (C.K.); 2 Collège Doctoral, Sorbonne Université, 75005 Paris, France

**Keywords:** quorum sensing, aminoglycosides, SOS response, *Vibrio cholerae*, bacterial signaling, antibiotic tolerance

## Abstract

Antibiotics are well known drugs which, when present above certain concentrations, are able to inhibit the growth of certain bacteria. However, a growing body of evidence shows that even when present at lower doses (subMIC, for sub-minimal inhibitory concentration), unable to inhibit or affect microbial growth, antibiotics work as signaling molecules, affect gene expression and trigger important bacterial stress responses. However, how subMIC antibiotic signaling interplays with other well-known signaling networks in bacteria (and the consequences of such interplay) is not well understood. In this work, through transcriptomic and genetic approaches, we have explored how quorum-sensing (QS) proficiency of *V. cholerae* affects this pathogen’s response to subMIC doses of the aminoglycoside tobramycin (TOB). We show that the transcriptomic signature of *V. cholerae* in response to subMIC TOB depends highly on the presence of QS master regulator HapR. In parallel, we show that subMIC doses of TOB are able to negatively interfere with the AI-2/LuxS QS network of *V. cholerae*, which seems critical for survival to aminoglycoside treatment and TOB-mediated induction of SOS response in this species. This interplay between QS and aminoglycosides suggests that targeting QS signaling may be a strategy to enhance aminoglycoside efficacy in *V. cholerae*.

## 1. Introduction

Many bacterial species secrete small diffusible signaling molecules to synchronize multicellular behaviors which allow them to adapt and survive in natural environments [1,2]. The most studied intercellular communication mechanism is quorum-sensing (QS), which monitors local population density [3,4]. QS is achieved via the production and detection of extracellular small molecules called autoinducers. At low cell density, autoinducers diffuse away, but at high cell density their concentration increases and triggers synchronization of gene expression in bacterial populations. Gram-negative bacteria are able to produce and detect several classes of autoinducers. Autoinducer 1 (AI-1) is a species-specific signaling molecule, while autoinducer 2 (AI-2), which is produced by Gram-negative and Gram-positive bacteria, is able to mediate both intra and interspecies QS communication [5,6].

**Figure 1:**
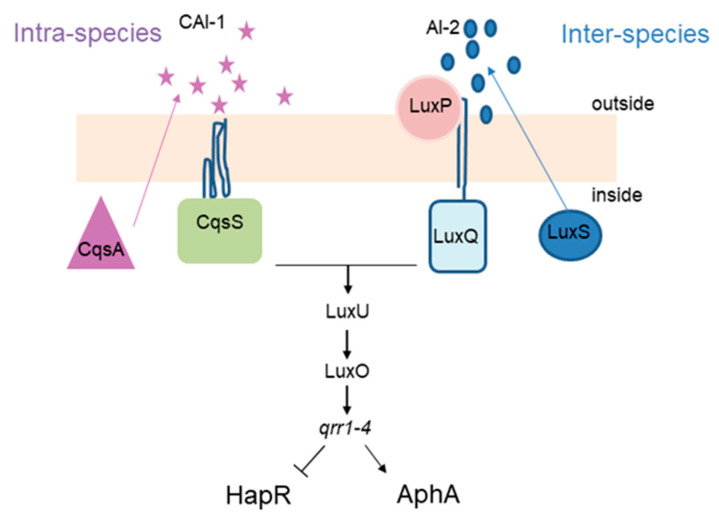
Simplified QS network of *V. cholerae*. Intra- and Inter-species autoinducers activate a phosphorylation cascade through LuxU and LuxO, leading to the expression of Qrr1–4 sRNAs which in turn promote expression of the AphA while inhibiting expression of HapR.

*Vibrio cholerae*, the causative agent of cholera disease, produces both autoinducers. AI-1, called cholera AI-1 (CAI-1), is produced by the CqsA protein and sensed by CqsS, while AI-2 is produced by LuxS and sensed by LuxQ, via LuxP periplasmic protein. The QS regulatory network of *V. cholerae* relies on a well described phosphorylation cascade (Figure 1). At low cell density, CqsS and LuxPQ work as kinases and phosphorylate LuxU which will then transfer the phosphate to the regulator LuxO. Phosphorylated LuxO will then trigger the expression of four small RNAs (qrr1–4) which in turn allow for translation of AphA (master regulator of low cell density), while inhibiting that of HapR, the LuxR family master regulator of high cell density in *V. cholerae*. By contrast, at high cell numbers, both CAI-1 and AI-2 accumulate and bind the cognate receptors CqsS and LuxPQ, which will now act as phosphatases and inhibit the phosphorylation cascade described above. This leads to an absence of qrr1–4 sRNAs and, consequently, the absence of AphA. Concomitantly, HapR is produced, inducing the expression of several genes involved in group behavior [7,8].

*Escherichia coli* can also detect autoinducers produced by other bacteria and react to them via SdiA, a LuxR protein homologue [9,10]. *E. coli* AI-2 is produced by the LuxS protein and sensed by the proteins encoded by the *lsr* operon [11,12].

Interestingly, bacterial communication through small molecule signaling can induce antibiotic tolerant phenotypes [13,14,15]. In parallel, it is also known that antibiotics at low doses can work as signaling molecules [16]. While studying the bacterial response to antibiotics, we showed that antibiotics from different families induce stress responses in Gram-negative bacteria, at concentrations below the minimal inhibitory concentration (subMIC), namely the SOS response [17,18]. SOS induction reflects the presence of a genotoxic stress to which the bacterial cell responds by triggering mutagenic DNA repair and recombination pathways, as well as rearrangements in the Superintegron carried by the *V. cholerae*’s second chromosome, which carries antibiotic resistance and adaptation genes [19,20]. We have pursued with the study of the response to aminoglycosides (AGs), which is a class of antibiotics that target the ribosome and induce mistranslation [21]. The AG-mediated SOS induction that we observed in *V. cholerae* is conserved among distantly related Gram-negative pathogens, such as *Photorhabdus luminescens* and *Klebsiella pneumonia* [18]. This observation was puzzling, because AGs do not directly target DNA synthesis or DNA molecules. Strikingly, we observed that the induction of SOS by low doses of AGs appeared to be dependent on HapR [17], because SOS induction by the aminoglycoside tobramycin was prevented in the *V. cholerae* strain lacking *hapR*. This observation suggested that QS could play a significant role in the evolution of antibiotic resistance.

We thus decided to study the impact of quorum sensing on the effect of sub-inhibitory concentrations of AGs in *V*. *cholerae*. We constructed mutants deriving from the N16961 HapR+ strain (referred to as wild-type), deleted for the genes *cqsA* (deficient for CAI intra-species signaling), *luxS* (deficient for AI-2 inter-species signaling), *luxPQ* (deficient for AI-2 sensing), *luxO* (“locked” in high cell density state) and *aphA* (the master regulator of low cell density). We asked which QS pathway(s) are involved in the response to sub-inhibitory concentrations of aminoglycosides, and how QS is involved in modulation of gene expression patterns by treatment with sub-inhibitory concentrations of AGs.

RNA-seq performed on both QS proficient (HapR+) and QS deficient (HapR−) *V. cholerae* strains points to major differences on global gene expression in response to subMIC tobramycin (TOB) treatment. Moreover, transcriptomic data suggest that subMIC AG treatment may interfere with the quorum sensing pathways and lead to the activation of the AphA low cell density regulon. We find that supplementation of growth media with AI-2 alleviates SOS induction by subMIC TOB. We further show that deletion of *luxS* (and to a lesser degree *cqsA*) is strongly detrimental for growth in presence of sublethal AGs concentrations and also for survival to lethal doses of this antibiotic family. These observations strongly suggest that QS signaling plays an important role in the response to antibiotics.

## 2. Materials and Methods

### 2.1. Bacterial Strains and Plasmids

Strains and plasmids are described in Table 1. Primers used in this work are listed in Supplementary Table S1. *V. cholerae* N16961 hapR+ derivatives were constructed by natural transformation as described [22]. Allelic replacements were performed using an assembly PCR fragment carrying 500 bp up and down regions of the gene to be deleted, and replaced by *aadA* spectinomycin resistance gene, using specified primers. Selection was performed using spectinomycin 100 µg/mL.

### 2.2. Tolerance Assays

Tolerance assays were performed on early stationary phase cultures. Overnight *V. cholerae* cultures were diluted 1000x in 10 mL fresh Mueller–Hinton (MH) medium and incubated at 37 °C with shaking. When cultures reached an OD_600_ 1.0, aliquots were serial diluted and spotted on MH plates. 3 mL of cultures were then collected into 14 mL Falcon tubes and treated with lethal doses of desired antibiotics (10 to 20 times the MIC: tobramycin 20 µg/mL and gentamicin 10 µg/mL), for 4 h at 37 °C with shaking in order to guarantee oxygenation. Serial dilutions were then spotted on MH agar without antibiotics. Experiments were performed 3 times.

### 2.3. MIC Determination Using Etests

Stationary phase cultures were diluted 20 times in PBS, and 300 µL were plated on MH plates and dried for 10 min. Etests (Biomérieux SA, Marcy-l’Étoile France) for Tobramycin and Gentamicin were placed on the plates which were then incubated overnight at 37 °C.

### 2.4. RNA-seq

Overnight cultures of the O1 biovar El Tor N16961 *hapR+* or *hapR− V. cholerae* strain were diluted 100x and grown in triplicate in MH medium until an OD_600_ of 0.4 with or without 0.02 µg/mL tobramycin. Sample collection, total RNA extraction, library preparation, sequencing and analysis were performed as previously described [24].

### 2.5. Growth Curves

Overnight cultures were diluted 100x in fresh medium, on 96-well plates. Each well contained 200 µL. Plates were incubated with shaking on TECAN device at 37 °C, OD_600_ was measured every 15 min. 

### 2.6. SOS Induction

SOS induction measurements by flow cytometry was performed as previously described [17,18,25]. Briefly, overnight cultures were diluted 100-fold in MH or MH supplemented with subMIC tobramycin (0.02 μg/mL), subMIC ciprofloxacin (0.005 μg/mL) and/or AI-2 (10 μM) and were incubated overnight at 37 °C. Fluorescence was then measured in 100,000 cells on the Miltenyi MACSquant device. The fluorescence values in each condition were normalized to the fluorescence values obtained in MH.

### 2.7. Luminescence Measurements in P. luminescens

Overnight cultures in Schneider media supplemented with 10 µM Na-borate, were diluted at OD_600_ 0.15 at grown at 30 °C. When cultures reached an OD_600_ of 0.9, 0.5 µg/mL tobramycin were added. After four hours, luminescence was measured as previously described [26]. Relative value was calculated: luminescence/OD_600_. Experiment was performed at least three times.

### 2.8. Statistical Analysis

Student’s t-test (unpaired) was performed using GraphPad Prism to determine the statistical differences between two groups. * indicates *p* < 0.05. Number of replicates for each experiment was 3 < *n* < 6. Means were also calculated using GraphPad Prism.

## 3. Results

### 3.1. Quorum Sensing Proficiency Influences the Response of V. cholerae to SubMIC Tobramycin

To have a global view on the gene expression patterns and their alterations by treatment with sub-inhibitory concentrations of tobramycin, we undertook a global study using RNA-seq. 

Since TOB at 2% of the MIC was previously shown to differently impact stress responses of *V. cholerae* in a QS proficient *hapR*+ strain [17] and in the N16961 strain carrying a frameshift inactivating the *hapR* gene, we performed RNA-seq in exponential phase cultures of *V. cholerae* of both strains treated or not with TOB at 2% of the MIC.

**Figure 2:**
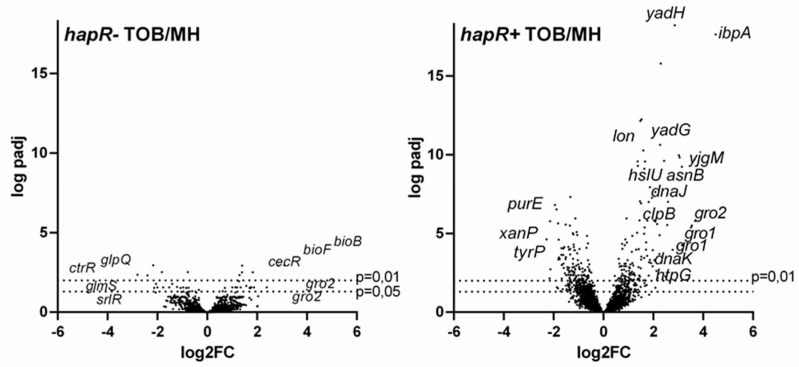
Transcriptomic profiles of *V. cholerae* cells treated with subMIC TOB depend on HapR proficiency. Volcano plots depicting gene expression changes caused by subMIC TOB (2% MIC) in HapR deficient (**left panel**) or HapR proficient (**right panel**) *V. cholerae* cells. The names of the genes with the strongest fold changes are represented. The x-axis represents the log_2_ of the fold change plotted against the log^10^ of the adjusted *p*-value.

Strikingly, we observed that the number of significantly differentially regulated genes by TOB in the HapR proficient strain was nearly twice the number of those differentially regulated by TOB in the *hapR−* strain: 366 and 566 genes were at least 1.5-fold up- and downregulated in *hapR+* against 259 and 238 genes in *hapR−*, respectively (Figure 2). Similarly, 70 genes with ≥ 3-fold change were found to be affected in *hapR+* strain, against 45 in *hapR−* (Figure 2).

First, looking at general effects of subMIC TOB on both strains, our analysis reveals the upregulation of chaperones and protein degradation factors, usually involved in the response to heat shock and protein stress (e.g., GroEL-ES, IbpA, Lon), showing that, even at doses that do not affect growth (here 50-fold lower than the MIC), TOB still yields protein stress. However, such stress seems to be more important in the *hapR*+ strain, as the induction of the two *groEL-ES* operons is 11- and 13-fold in *hapR*+ versus 3- and 4-fold for *hapR*−. Similarly, *ibpA* is induced 22-fold in *hapR*+ against 3-fold in *hapR*− (Supplementary Figure S1A).

Other categories of modulated gene expression include sugar metabolism and transport, as well as iron-related, genes. Markedly, sugar transport and metabolism genes are strongly downregulated by TOB in the *hapR*− strain (Supplementary Figure S1B). This effect is also found in the *hapR*+ strain, but to a lesser degree, which may be explained by the fact that the level of these RNAs is already lower in the *hapR*+ strain compared to *hapR*−.

Our second observation is that there are also dissimilarities between the two strains in pathways that are mobilized in response to TOB. There are major differences in translation (mostly downregulated by TOB in *hapR*+) (Supplementary Figure S1C) and iron-related genes (mostly up in *hapR*−) (Supplementary Figure S1D). Strikingly, ribosomal protein expression is oppositely modulated in the two strains (Supplementary Figure S1C). Since AGs target translation, reduction of translation in response to low levels of TOB can be an adaptive response, especially in *hapR+* strain. Importantly, the basal expression levels of ribosomal genes appear to be already higher in the absence of TOB in the *hapR+* strain and TOB reduces their expression to the *hapR*− levels. Iron- and energy-related genes are markedly increased by TOB in *hapR*−, while no major change was observed in *hapR*+ (Supplementary Figure S1D).

### 3.2. TOB Influences QS Response of V. cholerae and Interferes with AI-2 Signaling of Photorhabdus Luminescens

**Figure 3:**
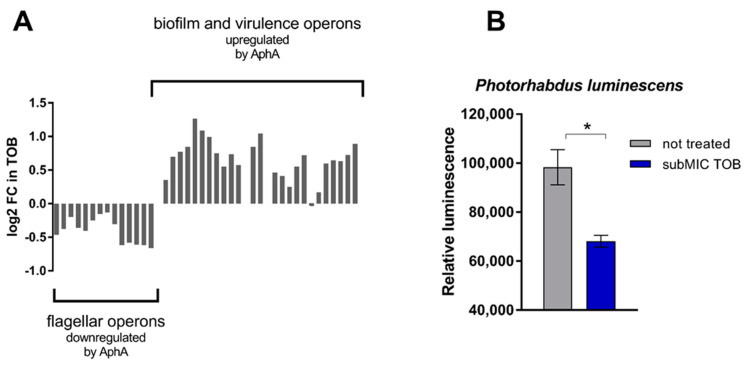
SubMIC TOB interferes with QS signalling. (**A**) Expression changes induced by subMIC TOB (2% MIC) of genes belonging to operons known to be regulated by AphA. (**B**) Effect of subMIC TOB (50% MIC) on QS-dependent luminescence of *P. luminescens*. Error bars indicate standard deviations. * *p*-value < 0.05 by Student’s *t*-test.

Regarding QS, we observed that genes known to be activated or inhibited by AphA [27,28] in *V. cholerae* are, respectively, up- and downregulated by TOB in *hapR*+ strain (Figure 3A), suggesting activation of the AphA low cell density regulon upon TOB treatment. In *V. cholerae*, the level of AphA protein is known to be negatively controlled by the concerted action of AI-2, CAI-1 and DPO (an autoinducer that is part of a third QS pathway in *V. cholerae* [29]), with AphA being barely detectable upon the simultaneous presence of these autoinducers [30]. However, eliminating AI-2 signaling is sufficient for AphA to be detected, even though at low levels [30]. Thus, in order to determine whether TOB has a positive or negative influence on AI-2 regulated phenotypes, we tested the effect of sublethal TOB treatment on bioluminescence in *Photorhabdus luminescens* [23], where natural luminescence is induced by elevated AI-2 levels [26]. We used an *uvrY* deficient strain as reference strain, as a previously shown decreased amount of AI-2 in this strain allows more sensitive measurements of AI-2 dependent luminescence, in comparison to the wild-type [26]. We found that subMIC TOB treatment significantly decreases luminescence in *P. luminescens* (Figure 3B). To prove that the negative effect of tobramycin on bioluminescence production was indeed due to targeting of the QS network of *P. luminescens* we performed the same experiment using a *luxS* deficient strain (Supplementary Figure S2). The results show that in absence of *luxS*, tobramycin no longer impacts bioluminescence production in *P. luminescens* (Supplementary Figure S2). Together with the activation of AphA regulon, these observations suggest that subMIC TOB interferes with AI-2 signaling and mimic a low-cell-density state.

### 3.3. AI-2 Signaling Alleviates SOS Response Induction by SubMIC TOB

**Figure 4:**
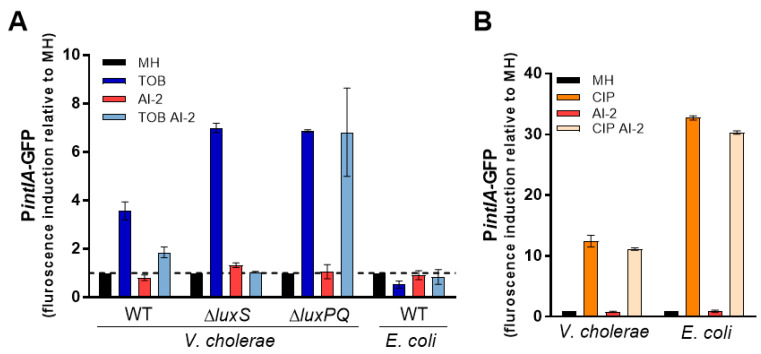
Aminoglycoside-mediated SOS induction in *V. cholerae* depends on LuxS/AI-2 signalling. SOS induction by (**A**) subMIC tobramycin (TOB, 0.02 μg/mL) or (**B**) subMIC ciprofloxacin (CIP, 0.005 μg/mL) in *V. cholerae* and *E. coli* cells.

We previously found that subMIC TOB induces SOS response in *V. cholerae*, but not in *E. coli* [17]. However, in *V. cholerae*, subMIC TOB only induces the SOS response in the *hapR+* but not in the *hapR*− strain, suggesting that QS is important for the effect of subMIC TOB ([17] and Supplementary Figure S2). Since bioluminescence data suggest modulation of *luxS* pathway by TOB, we asked whether the levels of AI-2 could have an effect on SOS response induction by TOB. We used GFP reporters of the SOS response regulated *intIA* promoter, as previously described [17], and confirmed that TOB induces SOS in *V. cholerae hapR+* but not in *E. coli* (Figure 4A). Interestingly, addition of exogenous AI-2 alleviates SOS induction by TOB, suggesting that AI-2 counteracts the effect of TOB. To confirm that this effect was specifically due to sensing of AI-2 by the bacterial cells, we constructed a strain deleted for the AI-2 sensor *luxPQ* operon. We confirmed that SOS response is also induced in the strain lacking *luxPQ* but the negative effect of AI-2 on TOB mediated SOS was lost. This is consistent with the hypothesis that sensing of AI-2 by *V. cholerae* minimizes the toxic effect of subMIC TOB. We also tested the effect of AI-2 on ciprofloxacin (CIP) induced SOS response in both *V. cholerae* and *E. coli* (Figure 4B). While both species induce SOS in response to CIP (as expected), we found no effect of AI-2. CIP induces SOS through direct DNA damage and our results show that AI-2 specifically interferes with subMIC TOB induced SOS, and does not prevent this induction by DNA damage. Furthermore, we constructed a *V. cholerae* strain deleted for *luxS*, and observed that SOS response induction by subMIC TOB increased in this strain, and addition of AI-2 was sufficient to counteract this effect (Figure 4A). Taken together, these data show that interspecies QS signaling interferes with the effect of subMIC TOB in *V. cholerae*.

### 3.4. AI-2 Signaling Improves Growth in SubMIC TOB and Tolerance to Lethal TOB Concentrations

**Figure 5:**
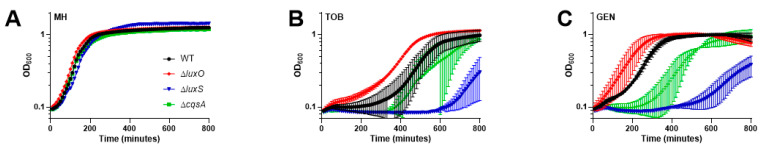
Growth curves of *V. cholerae* QS mutants in (**A**) absence or (**B**) presence of sublethal concentrations of tobramycin (TOB, 0.5 μg/mL) and (**C**) gentamicin (GEN, 0.5 μg/mL). Error bars indicate standard deviation.

We next tested the impact of QS on the susceptibility to antibiotics. We first tested the ability of QS mutants to grow in the presence of subMIC of the aminoglycosides TOB and gentamicin (GEN) (Figure 5). We used a higher subMIC (50% of the MIC) to assess growth of WT and different QS mutants: *cqsA* (deficient in CAI-1), *luxS* (deficient in AI-2 signaling) and *luxO* deletion mutant, which is “locked” in a high cell density state. In fact, the *luxO* deletion mutant mimics a state in which CAI-1 and AI-2 levels are high. We measured growth in microtiter plates by following the OD_600_ for 16 h. We found that deletion of *luxS* highly impacts growth in subMIC TOB and GEN (Figure 5B,C). As a corollary, deletion of *luxO* slightly improves growth in presence of both antibiotics. On the other hand, deletion of *cqsA* negatively impacts growth in presence of gentamicin but to a lesser extent when compared to the *luxS* mutant (Figure 5C). This suggests a protective role of AI-2 signaling against aminoglycoside action in *V. cholerae* cells.

**Figure 6:**
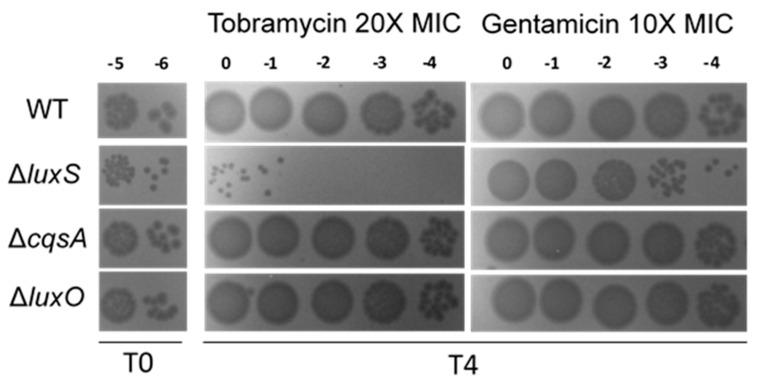
Tolerance of *V. cholerae* QS mutants to lethal concentrations of aminoglycosides. Bacterial populations were treated with lethal concentrations of tobramycin (20X MIC) and gentamicin (10X MIC) for 4 h. Represented are 10 μL drops of the indicated dilutions prior treatment (T0) and post treatment (T4). This is a representative experiment of three independent experiments with similar results.

We further asked whether deletion of *luxS* increases susceptibility to lethal doses of antibiotics (Figure 6). We observed no difference in the MIC of neither tobramycin nor gentamicin when we compared different mutants to wild type (Table 2). We thus treated *V. cholerae* cultures grown in rich media at OD_600_ 1.0 with these antibiotics at 10 to 20x the MIC for 4 h, and counted the proportion of surviving CFUs (Figure 6). Strikingly, when compared to the WT, the number of surviving bacteria is highly decreased in the ∆*luxS* strain (≈2 and 4 logs difference in gentamicin and tobramycin, respectively) with ∆*cqsA* and ∆*luxO* mutants exhibiting equivalent survival to WT strain.

## 4. Discussion

We based this study on our previous findings that SOS is induced by subMICs of aminoglycosides when QS proficient (*hapR*+) but not when QS deficient (*hapR*−) *V. cholerae* ([17] and Supplementary Figure S2). The observation that the *V. cholerae* strain lacking HapR fails to trigger aminoglycoside-mediated SOS induction prompted us to investigate how the responses to subMIC aminoglycosides vary in different QS contexts in this species. The transcriptomic analysis of both HapR− or HapR+ cells treated with 2% MIC of tobramycin revealed substantially different gene expression profiles between the two strains, specifically regarding the expression of genes involved in translation, cell energy and sugar transport processes (Supplementary Figure S1), which are known to modulate the physiological activity of aminoglycosides in bacteria [14,31,32,33,34]. Aminoglycosides are antibiotics known to target the ribosome, generating mistranslation and protein stress [35]. In agreement with this, we observed the induction of several members of the heat-shock regulon by subMIC TOB in both strains, showing that even very low concentrations of these drugs are able to generate protein stress in *V. cholerae*. However, the extent of this induction seems to be dependent on the QS state of the cells, as we noticed a greater induction of the heat-shock regulon in *hapR+* cells, thus suggesting a link between QS and response to aminoglycoside treatment in *V. cholerae*. Moreover, when we treated the *hapR*+ strain of *V. cholerae* with subMIC TOB we observed the upregulation of several genes whose expression is positively controlled by AphA, and the downregulation of genes known to be repressed by AphA [27,28] (Figure 3A). This suggests that subMIC tobramycin treatment mimics a state of low cell density, which is characterized by the absence (or low concentration) of autoinducers and the lack of activation of the respective QS systems. How subMIC TOB leads to the activation of the low cell density regulon is not clear, but it is possible that subMIC tobramycin interferes with one or several of these QS systems. In fact, we show that subMIC TOB seems to interfere with AI-2 QS signaling, as we observed that AI-2-dependent bioluminescence production in *Photorhabdus luminescens* is halted by subMIC TOB. Thus, by interfering with LuxS/AI-2 system, subMIC TOB could partially inhibit the LuxS–LuxO phosphorylation cascade and lead to an increase of AphA protein levels in the cell with the activation of AphA regulon. However, it has been suggested that the QS network of *V. cholerae* is quite robust, is resilient to signal perturbations by relying on four functionally redundant QS circuits [8], and that full QS network activation requires the concerted action of AI-2, CAI-1 and DPO molecules, which act together to fully repress AphA [30]. Thus, it is possible that some other additional factors, together with low AI-2 signaling, can be involved in the AphA regulon activation by subMIC TOB.

Given the lack of AG-mediated SOS induction in *V. cholerae hapR*− (Supplementary Figure S3), and the observation that AI-2 signaling seems to be affected by tobramycin (Figure 3B), we also sought to determine whether AG-mediated SOS induction in *V. cholerae* relies on this interspecies QS system. We found that subMIC TOB generates higher levels of genotoxic stress in absence of AI-2 signaling, as we observed a greater induction of SOS response in the *luxS* mutant (Figure 4). Moreover, deficiency of LuxS is highly detrimental for *V. cholerae* growth in subMIC aminoglycosides (Figure 5) and survival to lethal doses of these antibiotics (Figure 6).

The results described here suggest an interplay between aminoglycoside activity and QS in *V. cholerae*: on one hand, we show that subMIC TOB affects AI-2 signaling. On the other, we demonstrate the QS state of the cells (specially mediated by the luxS/AI-2 system) seems to dictate the response of *V. cholerae* to aminoglycosides. 

Several studies have demonstrated that QS signaling in bacteria often controls a multitude of processes that promote tolerance and resistance to several antibiotics. For example, expression of the MexAB-OprM efflux pump is positively controlled by QS and promotes resistance to beta-lactams in *Pseudomonas aeruginosa* [36]. Additionally, biofilm formation, which is critical to aminoglycoside susceptibility [37], is known to be controlled by QS [38,39], and it has been shown that the susceptibility of *P. aeruginosa* biofilms to aminoglycosides increases in presence of QS inhibitors of the LasI/LasR and RhlI/RlhR systems [40].

In addition, the AI-2/LuxS interspecies QS system has also been shown to modulate antibiotic resistance mechanisms in several species. Examples include the AI-2/LuxS—dependent upregulation of MDR efflux pumps, which promotes fluoroquinolone resistance in *E. coli* [41] and *Streptococcus suis* [42], or the AI-2/LuxS-dependent upregulation of a two-component system responsible for increasing vancomycin resistance in *Staphylococcus aureus* [43]. Other examples linking AI-2/LuxS QS system and drug resistance are reviewed in [44].

In *V. cholerae*, the molecular mechanisms behind AI-2-mediated protection against aminoglycosides remain to be elucidated. Nonetheless, such protection raises the interesting possibility that even small populations of *V. cholerae*, when in a high-cell-density multi-species context, can be less susceptible to aminoglycoside action. This may be of particular importance in the context of infections in the human gut, where AI-2-producing communities may help low loads of *V. cholerae* to survive aminoglycoside treatment.

In parallel, the fact that subMIC TOB interferes with QS signaling may have important consequences in the context of infection. In fact, AI-2 seems to be the necessary signal to repress biofilm formation and induce dispersal in *V. cholerae* [45]. Thus, by interfering with this QS system, low doses of aminoglycosides may enhance biofilm formation and virulence of *V. cholerae*. Further work is necessary to uncover the mechanism by which low doses of tobramycin (and potentially aminoglycosides in general) disrupt the interspecies QS system. Given that we do not observe any effect of TOB on the transcription of QS genes in our RNAseq data, one hypothesis may be that aminoglycosides affect the correct synthesis of specific proteins involved in this system. Alternatively, aminoglycoside molecules may directly interfere with AI-2 receptors. In fact, subMIC aminoglycosides attenuate QS-mediated virulence phenotypes in *P. aeruginosa*, and they have been found to possess strong binding properties to the QS receptor of *P. aeruginosa*, LasR [46].

Overall, the results obtained here contribute to the notion that QS communication and antibiotic resistance/tolerance mechanisms are linked. A link between bacterial signaling and antibiotic tolerance was also previously shown for a different signaling system, through indole secretion [13,15]. Manipulation of cell-to-cell signaling may thus be a potential way to fight antimicrobial resistance.

## Figures and Tables

**Table 1:** Strains and plasmids used in this study.

Strains and Plasmids	Strain #	Construction	
*E. coli* MG1655			Laboratory collection
*P. luminescens* TT01 *∆uvrY*	PL2105		[23]
*V. cholerae* N16961 *hapR−*	7805		Laboratory collection
*V. cholerae* N16961 *hapR+*	8637/F606		Laboratory collection
*V. cholerae* N16961 *hapR+* derivatives			
*luxO*(VC1021)::spectinomycin	J419	PCR assembly and natural transformation	ZIP413/414 and ZIP415/416 on gDNA. ZB47/48 on pAM34. Assembly ZIP413/416
*cqsA*(VCA0523)::spectinomycin	J422	PCR assembly and natural transformation	ZIP81/82 and ZIP83/84 on gDNA. ZB47/48 on pAM34. Assembly ZIP81/84
*luxS*(VC0557)::spectinomycin	J439	PCR assembly and natural transformation	ZIP87/88 and ZIP89/90 on gDNA. ZB47/48 on pAM34. Assembly ZIP87/90
*luxPQ*(VCA0736-VCA0737)::spectinomycin	F562	PCR assembly and natural transformation	ZIP191/192 and ZIP193/194 on gDNA. ZB47/48 on pAM34. Assembly ZIP191/194
*pTOPO-PintIA350-gfp* kanamycin	9192	350 bp region upstream of *intIA* was fused to *gfp*.	[22]

**Table 2:** MIC measured by *E-test* (μg/mL).

Strain	Tobramycin	Gentamicin
WT	1	1
Δ*luxS*	1	1
Δ*cqsA*	1	1
Δ*luxO*	1	1

## Data Availability

Accession numbers for RNAseq: the data for the RNAseq of the hapR− strain has been submitted in the GenBank Sequence Read Archive (SRA) under project number: PRJNA506714. For hapR+ strain, the RNAseq data have been deposited at GEO: GSE182561 and are publicly available as of the date of publication.

## Supplementary Materials

The following are available online at https://www.mdpi.com/article/10.3390/cells10113227/s1, Figure S1: Fold change values of several members of the heat-shock regulon induced by subMIC TOB in the different QS background contexts, Figure S2: subMIC TOB (50% MIC) fails to affect luminescence of a *P. luminescens* Δ*luxS* strain, Figure S3: SOS induction in both hapR− and hapR+ context of *V. cholerae* by sub inhibitory concentrations of tobramycin (TOB) or mitomycin C (MMC), Table S1: Primers used in this study.
