# Supplementary material for: Interplay between Sublethal Aminoglycosides and Quorum Sensing: Consequences on Survival in V. cholerae
Source: Cells. 2021 Nov 18;10(11):3227. doi: 10.3390/cells10113227 (PMC8621022; doi:10.3390/cells10113227)
Supplement: Supplementary file 1 [file cells-10-03227-s001.zip › cells-1426447-supplementary.pdf]

Article

Interplay between sublethal aminoglycosides and quorum sensing: consequences on survival in *V. cholerae*

André Carvalho [1,2,†], Evelyne Krin [1,†], Chloé Korlowski [1], Didier Mazel [1,*] and Zeynep Baharoglu [1,*]

Supplementary Materials

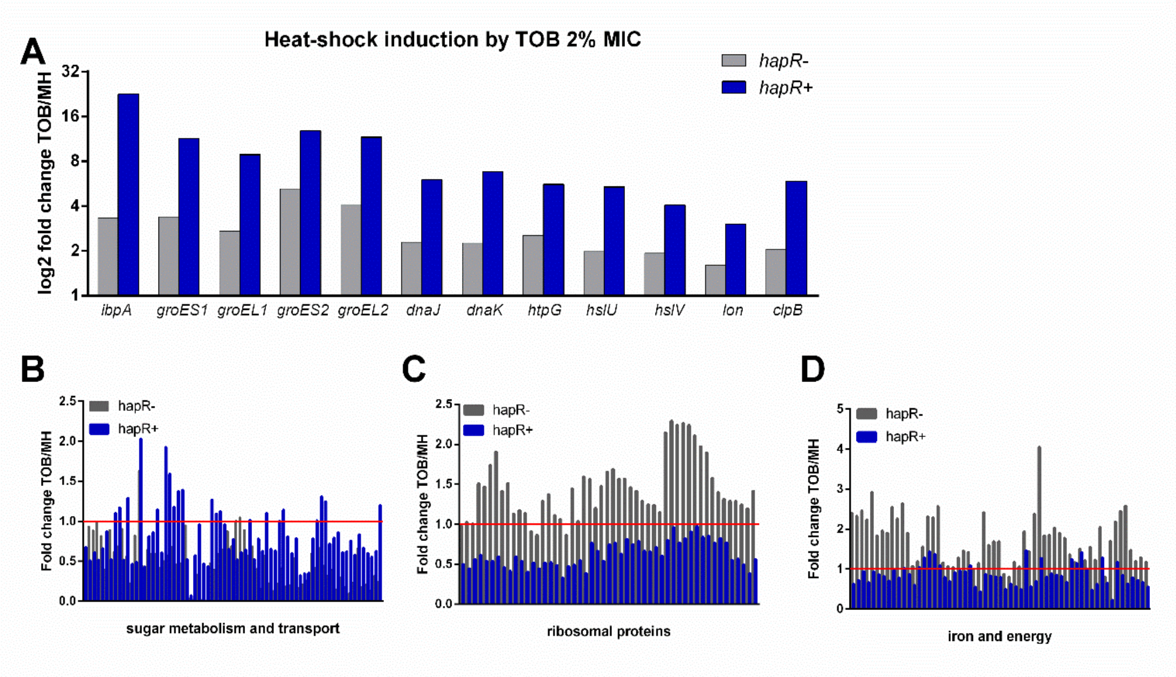

Figure S1. Fold change values of several members of the heat-shock regulon induced by subMIC TOB in the different QS background contexts.

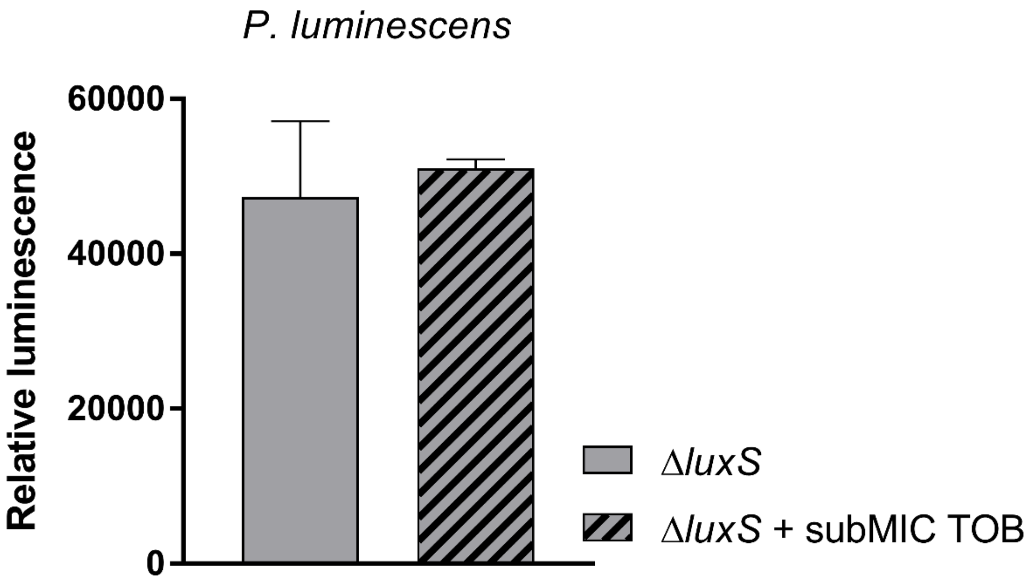

Figure S2. subMIC TOB (50% MIC) fails to affect luminescence of a *P. luminescens* ΔluxS strain.

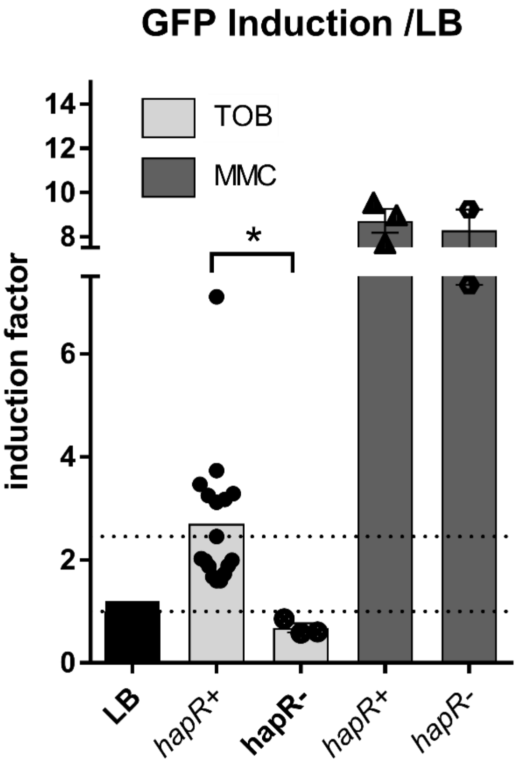

Figure S3. SOS induction in both hapR- and hapR+ context of *V. cholerae* by sub inhibitory concentrations of tobramycin (TOB) or mitomycin C (MMC).

Table S1. Primers used in this study.

Name	Sequence
ZB47	CCCGTTCCATACAGAAGCTGGGCGAACAAACGATGCTCGC
ZB48	GACATTATTTGCCGACTACCTTGGTGATCTCGCCTTTCACG
ZIP413	AAACGTGATGAAATTCATCAGTTGCG
ZIP414	GCGAGCATCGTTTGTTCGCCCAGCTTCTGTATGGAACGGGCACCGACGCCGTGTCTTCTACC
ZIP415	CGTGAAAGGCGAGATCACCAAGGTAGTCGGCAAATAATGTCGCGATGAGAGAATGGATCAACC
ZIP416	GAATATGGACCTGCAGGACGAATGAGC
ZIP81	GCCAACGATGGTAGTGAAGTGCTCAAGC
ZIP82	GCGAGCATCGTTTGTTCGCCCAGCTTCTGTATGGAACGGGATGCATAAATAACAAAAACTGCTAAACG
ZIP83	CGTGAAAGGCGAGATCACCAAGGTAGTCGGCAAATAATGTCCGCAATATATCCTAGTTATAAAAAATTTAACG
ZIP84	TGACAGGGGCTTTAGCGGGCATTGCC
ZIP87	AAGTGGTACTGGAAGGTCGCAAGC
ZIP88	GCGAGCATCGTTTGTTCGCCCAGCTTCTGTATGGAACGGGTTCCTTTCTCCCTTTCTGGCTCGC
ZIP89	CGTGAAAGGCGAGATCACCAAGGTAGTCGGCAAATAATGTCGTCGGTTCTGTAAACGTTAAAAATACAAAAGC
ZIP90	GGTACACCGCTTAACGTTCAGCTGC
ZIP191	CGTGACTGGAGATAAAACGCAGATCTGG
ZIP192	GCGAGCATCGTTTGTTCGCCCAGCTTCTGTATGGAACGGGAACAAGATTCAGCAGCACGTATTGC
ZIP193	CGTGAAAGGCGAGATCACCAAGGTAGTCGGCAAATAATGTCCTATCAGAAACAGCGGAGATATTAGC
ZIP194	TCACCAAACAGCGCCGATTCAACGG
